# Enumeration and Localization of Mesenchymal Progenitor Cells and Macrophages in Synovium from Normal Individuals and Patients with Pre-Osteoarthritis or Clinically Diagnosed Osteoarthritis

**DOI:** 10.3390/ijms18040774

**Published:** 2017-04-05

**Authors:** Kate O’Brien, Pankaj Tailor, Catherine Leonard, Lisa M. DiFrancesco, David A. Hart, John R. Matyas, Cyril B. Frank, Roman J. Krawetz

**Affiliations:** 1 McCaig Institute for Bone & Joint Health, University of Calgary, Calgary, AB T2N4N1, Canada; icehouse.obrien@gmail.com (K.O.); ptailor@ucalgary.ca (P.T.); cleonard@ucalgary.ca (C.L.); hartd@ucalgary.ca (D.A.H.); jmatyas@ucalgary.ca (J.R.M.); cfrank@ucalgary.ca (C.B.F.); 2 Department Cell Biology and Anatomy, University of Calgary, Calgary, AB T2N4N1, Canada; 3 Department of Pathology & Laboratory Medicine, University of Calgary, Calgary, AB T2N4N1, Canada; Lisa.DiFrancesco@cls.ab.ca; 4 Department of Surgery, University of Calgary, Calgary, AB T2N4N1, Canada; 5 Department of Comparative Biology and Experimental Medicine, University of Calgary, Calgary, AB T2N4N1, Canada; 6 The D-BOARD European Consortium for Biomarker Discovery, School of Veterinary Medicine, Faculty of Health and Medical Sciences, University of Surrey, Guildford GU2 7XH, UK

**Keywords:** adult progenitor cells, osteoarthritis, macrophage, synovium

## Abstract

Osteoarthritis (OA) is a degenerative disorder characterized by chondrocyte apoptosis and degeneration of articular cartilage resulting in loss of mobility and pain. Inflammation plays a key role in the development and progression of OA both on the side of apoptosis and repair, while its exact role in pathogenesis has yet to be fully elucidated. Few studies have examined the cellular composition (inflammatory cells and/or progenitor cells) in the synovium of patients with pre-OA (asymptomatic with cartilage damage). Therefore, in the current study, mesenchymal progenitor cells (MPCs) and macrophages were enumerated within normal, pre-OA and OA synovium. No differences were observed between MPCs in normal vs. pre-OA, however, fewer macrophages were observed in pre-OA vs. normal synovium. Osteoarthritic synovium contained greater numbers of both MPCs and macrophages. Interestingly, the localization of MPCs and macrophages was affected by disease severity. In normal and pre-OA synovium, MPCs and macrophages co-localized, while in OA synovium, MPCs and macrophage populations were spatially distinct. Examining the cellular interactions between MPCs and macrophages in synovium may be essential for understanding the role of these cells in the onset and/or pathogenesis of the disease. This study has provided a first step by examining these cell types both spatially and temporally (e.g., disease severity). Further cellular and molecular studies will be needed to determine the functions of these cells in the context of disease and in relation to each other and the joint as a whole.

## 1. Background

Osteoarthritis (OA) is a degenerative joint disorder that affects all tissues in synovial joints [1,2,3]. Symptomatically, OA is characterized by joint pain and stiffness with eventual deformity and loss of mobility [4]. During the onset of OA, it has been demonstrated that articular chondrocytes initiate apoptotic cascades which eventually leads to alterations in articular cartilage composition, structure, and function [5]. Pathophysiologically, both cartilage degeneration and accelerated bone turnover have been identified as causal elements in the progression of OA [2]. Risk factors for OA include age, obesity, and previous joint trauma; however, the interactions among these factors is unknown [6,7,8,9]. Another tissue commonly affected in the pathogenesis of OA is the synovium. Synovium is the soft tissue lining the spaces of diarthrodial joints and contains highly metabolically active cells called synoviocytes that are further separated as macrophage-like (Type A) and fibroblast-like (Type B) synoviocytes. These cell types and the synovium as a whole play a key role in normal joint physiology as they facilitate nourishment for chondrocytes and the removal of waste products through the synovial fluid [10]. Inflammation of the synovium, known as synovitis, is commonly found in the early stages of OA, and while a number of animal and clinical studies have found a correlation between synovitis and disease severity and/or progression others have not [11,12]. However, because of the synovium’s influential role in maintaining the health of the joint, it is thought that this chronic inflammation of the synovium (and/or alternation of normal synovial function) is detrimental to the overall health of the joint itself.

Although historically OA was considered a degenerative disorder, more recently it has been proposed that OA is significantly more complex than previously thought. Ayral et al. performed a systematic review in which they stated that the processes involved in OA are seen as dynamic biological and biochemical processes [9]. Of significant importance in OA pathology is the interaction of articular cartilage and synovium [9,13,14]. Osteoarthritis-related synovitis results in a positive feedback loop: (a) cartilage breakdown releases tissue and molecular fragments into the synovial fluid; (b) phagocytes/macrophages in the synovium remove these fragments, inducing further signaling that promotes further synovial inflammation. The newly activated synoviocytes release catabolic and pro-inflammatory cytokines (including interleukin (IL)-1 and tumor necrosis factor-α (TNF-α)), which can lead to excess production of proteolytic enzymes (matrix metalloproteinases (MMPs); a disintegrin and metalloproteinase with thrombospondin motifs (ADAMTS) and others) and to remodeling of the matrix, as well as triggering apoptosis in chondrocytes and thereby further effect cartilage breakdown. Additionally, osteoarthritic synovium produces fewer antagonists of the IL-1 receptor, intensifying the catabolic effect of the above inflammatory cytokines in OA [15]. As cartilage breaks down further, the cycle continues [5,15]. Synovitis is thought to be responsible for many of the clinical symptoms of OA and has been shown to reflect the structural progression of the disease [5]. Clinically, synovitis is rarely observed without cartilage degradation [5], further solidifying the link between cartilage pathology and synovitis in OA.

As mentioned, the synovium is comprised of Type B synovial fibroblasts which produce lubricating molecules such as Hyaluronic acid (HA) and lubricin, but this population of cells also contains a sub-population of mesenchymal stem/progenitor cells (MSC/MPC), which are believed to be an important part of regeneration and healing in multiple tissues types. In OA, synovial MSCs/MPCs increase in number but demonstrate decreased chondrogenic potential [16]. Synovial macrophages are a likely regulator in this phenomenon, as it was recently found that MSC chondrogenesis can be inhibited by pro-inflammatory macrophages [17,18]. This suggests a relationship between degeneration (apoptosis) and repair in the joint with inflammation potentially acting as the intermediary. However, to date, there is no direct evidence in humans that cartilage can repair itself once injured/damaged, furthermore, a recent study has suggested that cartilage has little to no turnover after skeletal maturity regardless of the disease state of the individual (OA vs. normal) [19]. Therefore, it is necessary to determine the role of these MSCs/MPCs and macrophages in normal and arthritic synovium, and a logical first step is to enumerate these cells throughout the stages of OA. It is also important to note that we chose to use the terminology mesenchymal progenitor cell (MPC) instead of mesenchymal stem cell (MSC). This was done intentionally, since the name MSC is defined by the functional properties of the cell and not solely by marker expression. A histological examination of the spatial and temporal distribution and quantity of marker cannot define the functional capacity of a cell.

Previously, Hermida-Gomez et al. [20] quantified cells expressing MSC/MPC markers in healthy and OA synovium from hip joints and found that cells positive for MSC/MPC markers were increased in OA compared to normal synovium. This result is consistent with other findings [16,21], that suggest an increase in stem/progenitor cells with severity of cartilage damage/OA. However, no study to date has evaluated the differences in MPC levels in pre-OA versus normal knee synovium, and furthermore, to our knowledge no study has examined the localization of MPCs in relation to synovial macrophages throughout disease progression. In the current study, we therefore sought to examine if synovial MPCs and macrophage populations are also increased in pre-OA since it has been previously hypothesized that, as the severity of OA increases, so does the number of MPCs and immune cells present within the synovium. We have attempted to address this hypothesis by quantifying the number of cells expressing markers of MPC and macrophages present in normal, pre-OA, and OA synovium. Additionally, the localization of the cells (intima vs. subintimal) and overall inflammatory state of the tissue was also examined.

## 2. Results

### 2.1. Normal Synovium

**Figure 1:**
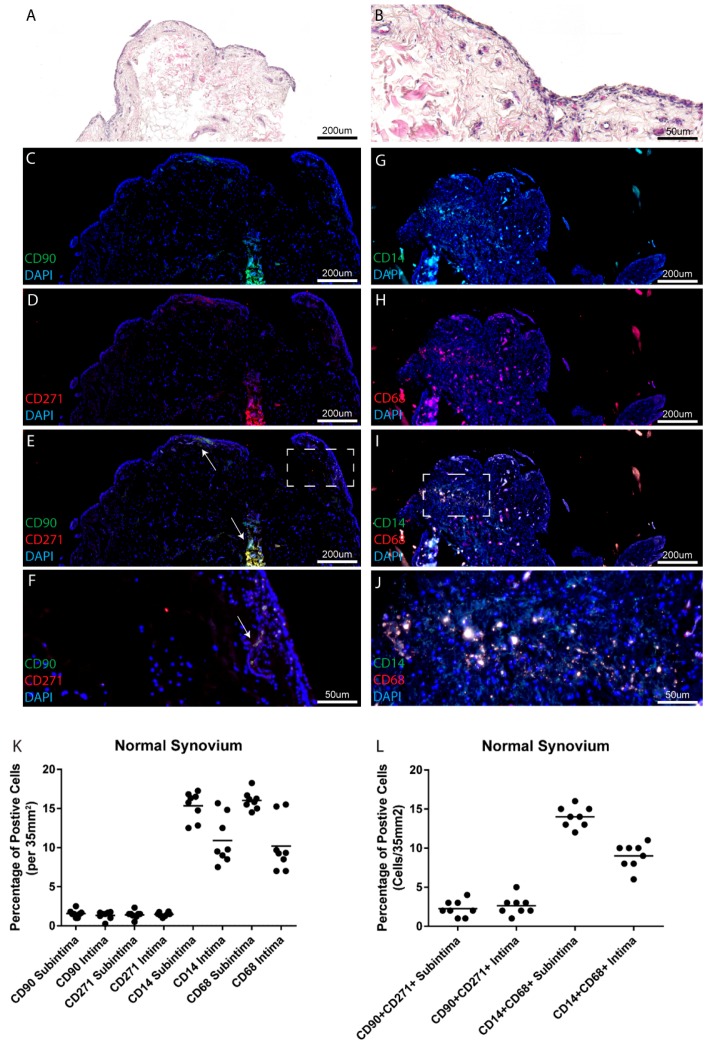
Mesenchymal progenitor cells (MPCs) and macrophages in normal synovium. Normal synovial samples from eight individuals (one patient shown as example), were examined histologically for tissue architecture with hematoxylin and eosin (H&E) staining (**A**,**B**) and for markers of MPCs (CD90, CD271) (**C**–**F**) or macrophages (CD14, CD68) (**G**–**J**). No differences were observed in the total numbers of MPCs in the intima or subintima within the normal knee joints examined at the level of single (**K**) or double positive cells (**L**), while a decrease in the number of CD14 and CD68 positive cells was observed in the intima vs. subintima. DAPI: 4′,6-diamidino-2-phenylindole.

Within the eight normal joints examined in this study, limited synovial inflammation was observed in the locations from which the biopsies were recovered (Table 1 and Figure 1A,B). Mesenchymal progenitor cells (CD90, CD271) (Figure 1C–F) and macrophage (CD14, CD68) (Figure 1G–J) marker positive cells were observed both in the intimal and subintimal tissue layers in all individuals (Figure 1C–J, example from one patient). When comparing biopsies collected from the normal joint cohort at the intimal and subintimal tissue layers, no differences were observed in putative MPCs (CD90 or CD271 positive cells) (Figure 1K), while decreases in putative macrophages (CD14 or CD68 positive cells) were observed in the intimal layer compared to the subintimal layer (Figure 1K). Overall, in the normal synovium, a low level of heterogeneity was observed between patients as can be observed within the quantified data (Figure 1K), and also within representative IF images from the remaining seven individuals (Figures S1 and S2). Since none of the markers employed in this study definitively identify either MPCs or macrophages, cells positive for both markers of each cell type were enumerated (Figure 1L). The examination of double positive cells was consistent with the number of single positive cells of each type, suggesting that the majority of CD90 positive cells were also positive for CD271 (75.2% ± 3.4%) and likewise the majority of CD14 positive cells were positive for CD68 (86.7% ± 3.1%). To test if this was the case, the number of single positive cells of each marker was compared to the number of double positive cells and it was observed that while single positive populations were present in normal synovium, the double positive cells accounted for the majority of the cells staining positive for each individual marker of MPCs or macrophages (Figures S3 and S4).

### 2.2. Pre-Osteoarthritis Synovium

**Figure 2:**
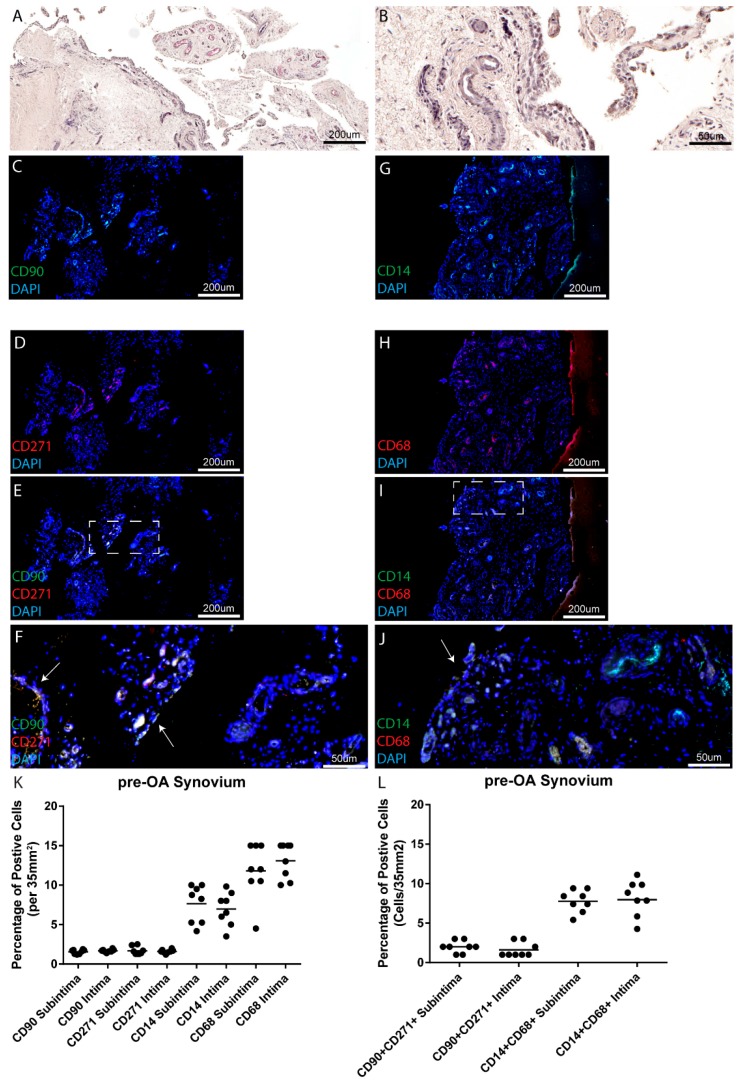
MPC and macrophage markers in pre-osteoarthritis (pre-OA) synovium. Pre-OA synovial samples from eight individuals (one patient shown as an example), were examined histologically for tissue architecture with H&E staining (**A**,**B**) and for markers of MPCs (CD90, CD271) (**C**–**F**) or macrophages (CD14, CD68) (**G**–**J**). No differences were observed in the total numbers of MPCs in the intima or subintima within the knee joints examined (**K**), however, decreased levels of CD14^+^ vs. CD68^+^ cells were detected in the intima and subintima. Double positive cells were also enumerated and no differences in cell number was observed in MPCs or macrophages between the subintimal vs. intimal tissue layers (**L**).

Within the eight pre-OA joints examined, moderate to high levels of synovial inflammation based on the Krenn synovitis score were observed at the areas biopsies were recovered from (Table 1 and Figure 2A,B). As in normal synovium, the MPC and macrophage markers examined in this study were observed in the intimal and subintimal tissue layers of all patients examined (Figure 2C–J, example from one patient) (representative images from remaining patients in Figures S1 and S2). When comparing biopsies collected from the pre-OA cohort at the intimal and subintimal tissue layers, no differences were observed in putative MPCs (CD90 or CD271 positive cells) (Figure 2K), however, unlike normal joints where a decrease in putative macrophages (CD14 or CD68 positive cells) was observed in the intimal layer compared to the subintimal layer, no differences in macrophage number (either CD14 or CD68 positive cells) were observed between the intimal and subintimal layers (Figure 2K). However, it was observed that fewer CD14 positive cells were observed in the intimal and subintimal layers compared to CD68 positive cells. As with the normal synovium, analysis of the double positive cells demonstrated that the majority of the cells positive for one marker were also positive for the other marker for either MPCs (71.1% ± 4.1%) or macrophages (76.1% ± 4.3%) (Figures S3 and S4). Furthermore, enumeration of the double positive cells (either MPCs or macrophages) also demonstrated no differences between the intimal and subintimal tissue layers (Figure 2L). However, because of the lower number of CD14 positive cells observed in both tissue layers, we observed a decrease in the number of CD14^+^ CD68^+^ double positive macrophages compared to the number of CD68^+^ single positive cells in both the intima and subintima layers.

### 2.3. Osteoarthritis Synovium

**Figure 3:**
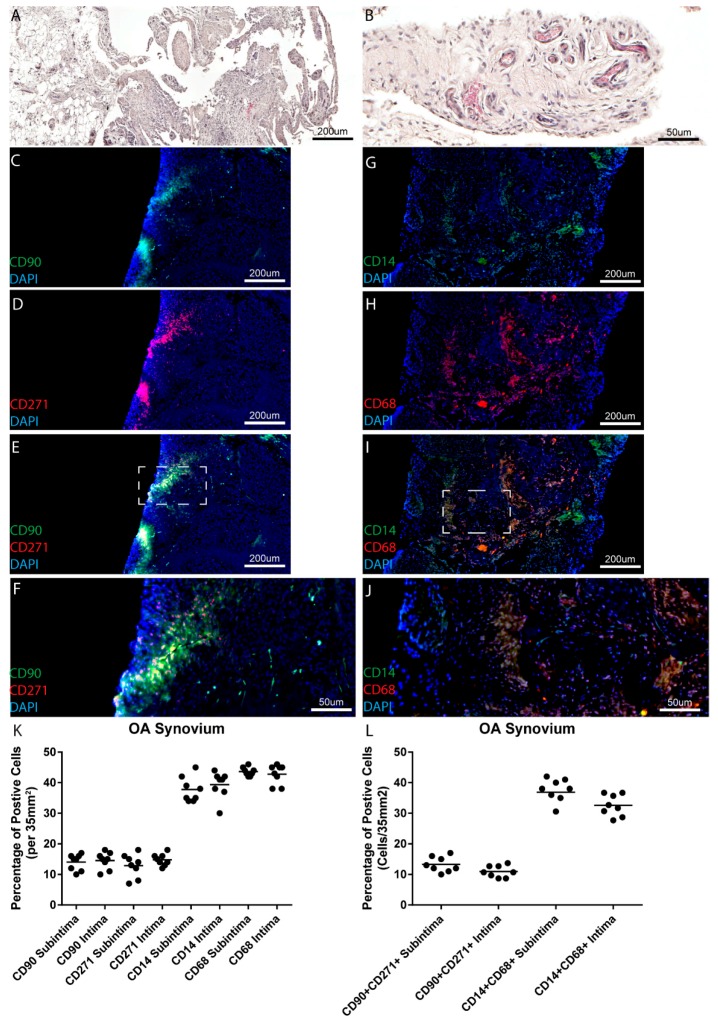
MPC and macrophage markers in OA synovium. Synovial samples from eight individuals diagnosed with OA (one patient shown as an example), were examined histologically for tissue architecture with H&E staining (**A**,**B**) and for markers of MPCs (CD90, CD271) (**C**–**F**) or macrophages (CD14, CD68) (**G**–**J**). No differences were observed in the total numbers of MPCs or macrophages in the intima or subintima within the knee joints examined (**K**,**L**).

Since it has been demonstrated in previous studies that the number of macrophages and MPCs increase with disease severity, a clinically diagnosed OA patient cohort was also examined in this study. Within the eight OA joints examined, high levels of synovial inflammation was observed in the areas from where the biopsies were recovered (Table 1 and Figure 3A,B). As in normal and pre-OA synovium, the MPC and macrophage markers examined were observed in both the intimal and subintimal tissue layers of all patients examined (Figure 3C–J, example from one patient) (representative images from remaining patients in Figures S1 and S2). No differences were observed in the number of putative MPCs or macrophages between the intimal vs. subintimal tissues layers in the OA patient cohort (Figure 3K). When the double positive cells were enumerated, no differences in either cell type was observed between the intimal vs. subintimal tissue layers (Figure 3L). When the different sub-populations were examined by marker expression, it was observed that 68.7% ± 4.2% of CD90 or CD271 positive cells were positive for both markers, while 65.9% ± 2.3% of CD14 or CD68 positive cells were positive for both markers.

### 2.4. Between Joint Findings: Normal vs. Pre-Osteoarthritis and Osteoarthritis

**Figure 4:**
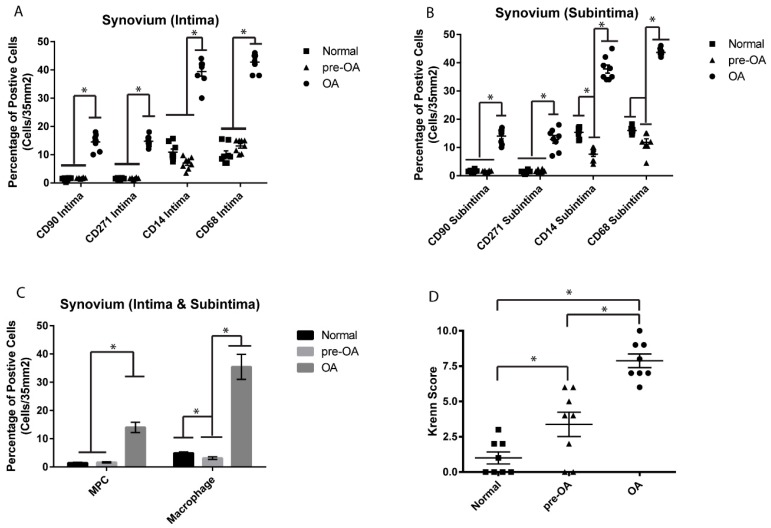
Comparison of MPCs and macrophages between normal, pre-OA and OA synovium. No difference was observed in the number of CD90 or CD271 positive cells in the intima or subintima between normal and pre-OA knee synovium (**A**,**B**), however, decreased numbers of CD14 positive cells were observed in the subintima of pre-OA vs. normal joints. No difference in MPCs (CD90^+^ CD271^+^) was observed between normal and pre-OA synovium (**C**), while fewer macrophages (CD14^+^ CD68^+^) were observed in pre-OA synovium (**C**). Furthermore, significantly more MPCs and macrophages were observed in the synovium (intima and subintima) of OA joints compared to both normal and pre-OA (**A**–**C**). The level of synovial inflammation increased with severity of the disease (**D**). * *p* < 0.05.

The cellular composition (MPCs and macrophages) of all normal pre-OA and OA synovial biopsies was examined to determine if any significant differences existed within the normal, pre-OA and OA cohorts in the intima (Figure 4A), subintima (Figure 4B) or synovium as a whole (Figure 4C). While no significant differences were observed in the number of CD90^+^ or CD271^+^ cells between normal vs. pre-OA, there were fewer CD14^+^ positive cells in the subintima (Figure 4B). When the synovium was examined as a whole using only double positive cell populations (CD90^+^ CD271^+^ or CD14^+^ CD68^+^), it was observed while there was no difference in MPCs between normal and pre-OA, there were significantly fewer macrophages in the pre-OA vs. normal synovium. When normal and pre-OA cohorts were compared to OA cohorts, there were significantly greater numbers of MPCs and macrophages observed in the intima, subintima and whole synovium in OA compared to the other two cohorts (Figure 4A–C). The result of fewer macrophages detected in the pre-OA cohort is in some ways contradictory to the total synovial inflammation in this cohort, particularly when the synovitis (Krenn) score was compared between normal and pre-OA cohorts, and it was observed that the pre-OA synovium presented with significantly increased synovitis compared to normal (Figure 4D).

### 2.5. Between Joint Findings: Cellular Localization in Normal vs. Pre-Osteoarthritis and Osteoarthritis

**Figure 5:**
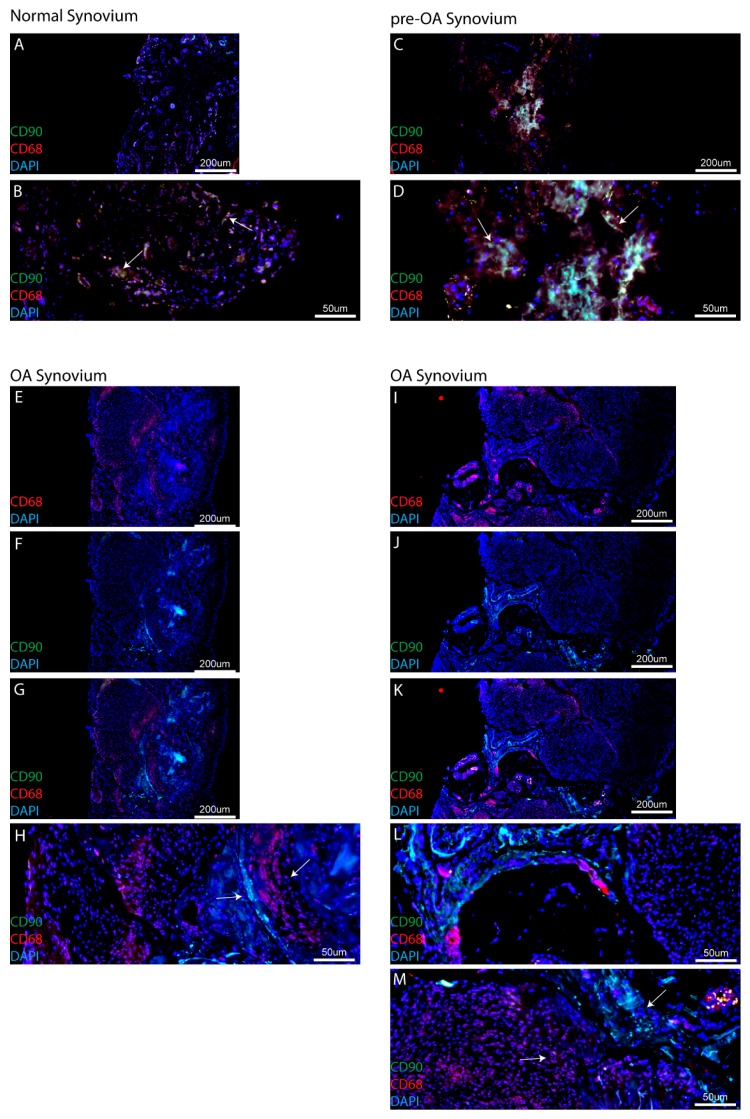
MPC and macrophage localization in synovium. In both normal (**A**,**B**) and pre-OA (**C**,**D**) synovial samples, MPCs (CD90) and macrophages (CD68) are observed in close proximity to each other (**B**,**D**, arrows). However, in OA synovial samples from two patients (**E**–**H**,**I**–**M**, as representative examples) there is a clear spatial separation of MSCs and macrophages (**H**,**M**, arrows).

Although MPC and macrophage populations were not found to be abundant in synovial biopsies from normal and pre-OA cohorts, it was observed during our analysis of serial sections that MPC and macrophage populations were typically observed in close proximity to each other, while in OA samples, it appeared that the populations were always spatially distinct within the synovium. However, since this was observed from serial sections and not within the same section, staining with one MPC marker (CD90) and one macrophage marker (CD68) was undertaken to examine this observation in more detail. Within synovial samples collected from the normal cohort, while only few MPC and macrophage cells were typically observed, it was found that in many cases these two cell population were found within close proximity to each other (Figure 5A,B arrows). This observation was not only limited to the normal synovial samples, as it was also found that in the synovium of patients with pre-OA, MPCs and macrophages resided in similar areas of the tissue (Figure 5C,D arrows). However, when biopsies from patients with OA were examined, a clear spatial separation between MPCs and macrophages was observed in all biopsies examined from this patient cohort (Figure 5E–H,I–M). Furthermore, in all the eight samples of OA synovium examined, no clear intermixing of CD90^+^ and CD68^+^ cell populations was observed.

## 3. Discussion

A number of previous studies have demonstrated that synovial MSC/MPC populations increase in OA. In the majority of these studies, a normal/control group was compared to a clinically diagnosed (typically end stage) OA patient cohort. While the results of the current study agree with previous finding between normal and OA joints, no increase in MPCs was observed in a pre-OA patient population that presented with cartilage damage and synovial inflammation, yet were asymptomatic and demonstrated no radiographic changes associated with OA. Furthermore, the same trend was observed with synovial macrophages between normal and OA knee synovium, however, fewer macrophages were observed in pre-OA patients compared to normal controls. The results and limitations of this study will be discussed in relation the published literature below.

In this study, we chose to examine the MSC/MPC markers CD90 and CD271 for a number of reasons. Primarily, both our group and others have demonstrated that synovial cells purified based on the basis of CD90^+^ demonstrated increased chondrogenic potential compared to the CD90^−^ population [22,23,24]. Additionally, it has been previously demonstrated in hip synovium that the CD90 ^+^ CD271^+^ double positive population is not only present throughout the synovium (intima and subintima) [25], but when CD271^+^ or CD271^−^ bone marrow-derived cells were used to treat a chondral defect, the CD271^+^ positive population demonstrated significantly increased repair potential [25]. While CD90 and CD271 are promising markers to identify MSC/MPC populations from synovium and other tissues, there are many additional marker normally used to characterize these cell population including but not limited to CD44, CD73, CD105, CD146. In this study, the main limitation was that we were not able to perform co-localization with more markers; however, that being said, it is widely agreed upon that the marker expression of a cell does not correlate to function, and to determine if a cell is truly an MSC, functional analysis must be undertaken. Therefore, in this study we have defined these cell populations as progenitor cells based on the previous literature demonstrating that synovial cells selected for by CD90 and/or CD271 meet the functional definition of a progenitor. However, it is important to be clear that although we have enumerated these cell populations, there is no guarantee that all CD90^+^, CD271^+^ or CD90^+^ CD271^+^ positive cells represent the same cell type and/or demonstrate a conserved function. Additionally, it is more than likely that the cells (MPCs and/or macrophages) examined in this study represent only a sub-population of a larger population that might not be accurately represented by the markers selected for this study.

Increased numbers of MSCs/MPCs have been found in OA joints as compared to normal joints, in both synovial fluid and the synovium itself, findings which have been confirmed in this study. Several studies have examined the quantity of stem cells found in synovial fluid. Jones et al. compared OA synovial fluid with rheumatoid arthritis (RA) synovial fluid and found increased numbers of MSCs in the OA synovial fluid [26]. Quantification revealed that MSCs were increased ten-fold in OA as compared to RA synovial fluid. It is unknown whether these MSCs are from the synovium itself or whether they could be recruited from bone, periosteum or bone marrow. Jones et al. [27] explored the above idea when they examined MSC in normal and OA human joints. The study was unique in that it examined synovial fluid in early OA, rather than late stage OA. They attempted to understand whether normal synovial fluid has a resident stem cell population, or whether an increase in stem cells in the fluid was directly linked to the disease state of the joints. After identifying the cell signature of the endogenous synovial fluid cells, they determined that the MSCs likely came directly from the synovial fluid [27].

Similarly, Sekiya et al. found that synovial fluid MSC increased as the severity of OA increased [21], and Hermida-Gomez et al. [20] found that there were significantly more CD44 and CD90 positive cells in OA synovium than in normal synovium. This effect was reversed, however, regarding cells positive for CD73; healthy synovium has greater numbers of CD73 positive cells than OA synovium [20]. This is interesting to note, considering that the presence of CD44, CD90 and CD73 all indicate MSCs. However, it has been shown using clonal analysis in vitro that multiple distinct MSCs populations reside within a given tissue. Therefore, one plausible hypothesis is that specific populations of MSCs were influenced by the disease state of the synovium and/or joint as a whole. Singh et al. characterized normal knee synovium using immuno-histochemistry (IHC). After removing synovial biopsies from 12 healthy subjects, samples were stained and analyzed for lymphocytes (T cells and B cells) and macrophages [28]. Findings indicated the presence of T cells and macrophages, but no B cells. Osteoarthritic tissue containing increased numbers of macrophages has been put forth as a mechanism by which endogenous MSCs in OA joints become dysfunctional [17]. Benito et al. [29] compared early stage OA synovium to late stage OA synovium. Findings showed that there were increased numbers of macrophages in the early OA synovium as compared to the late stage OA synovium. Macrophages in synovium contribute to the overall inflammatory cascade seen in OA. Products of cartilage breakdown are phagocytosed by the synoviocytes (macrophage-like), which inflames the synovium. Production of proinflammatory cytokines then amplifies this effect. Activated synovial T cells, B cells and macrophages increase the inflammatory response and contribute to a positive feedback loop [10]. In addition, macrophages themselves produce growth factors such as vascular endothelial growth factor and inflammatory cytokines such as IL-1B and TNF-a [30]. While in the current study, increased numbers of CD68^+^ CD14^+^ cells were observed in normal vs. OA synovium, however, OA synovium presented with a higher inflammatory score. Since CD68 and CD14 do not describe if a macrophage is M1 vs. M2 polarized, it is therefore possible that the CD68^+^ CD14^+^ cells detected in normal synovium may be M2 anti-inflammatory macrophages that are lost during the onset and progression of OA; however, further study with specific polarization markers will be needed to address this hypothesis. Additionally, we cannot discount the possibility that other immune cells and/or synovial fibroblasts are contributing to the increased synovial inflammation observed in the pre-OA cohort. Furthermore, it is also quite possible that pre-OA patients might have been self-medicating with various pharmacological agents (none of the pre-OA cohort were on prescription anti-inflammatories) which led to a decrease in macrophage/monocytic cells in this cohort.

The results of the current study using immunofluorescence staining showed the presence of both MPCs and macrophages. There was no correlation between synovial inflammation in pre-OA and increased numbers of the cell types examined. Additionally, there was no significant difference between the apparent cellularity of pre-OA synovium and normal synovium. These findings conflict with much of the current literature. Sekiya et al. [21] and Jones et al. [26] found increased numbers of stem cell type cells present in the synovial fluid of OA patients as compared to normal controls. The Hermida-Gomez study described above illustrated increased numbers of stem cells in OA synovium as compared to normal control synovium [20]. This being said, most of the existing studies were performed on late stage OA synovium or exclusively on synovial fluid. To date, there has been no study which has compared MPCs in pre-OA synovium to normal controls. However, in this study, when an OA cohort was examined (radiographic OA with confirmed cartilage changes during arthroscopy) increased numbers of both MPCs and macrophages were observed consistent with the published literature. Therefore, there could be a number of potential reasons explaining the findings in the current study. As we aimed to quantify cells found in synovium at the earliest stages of OA, most of the pre-OA patients used in this study demonstrated very low levels of synovitis in the joint (although increased compared to normal joints, but decreased compared to OA joints). The inflammatory threshold for cellular infiltration has not been established and therefore there may not have been sufficient inflammation to lead to increased cellularity. In this study, ‘pre-OA’ was asymptomatic. Considering that synovitis is typically associated with pain, this could indicate that subclinical OA synovium has few differences from ‘normal synovium’. As there have been no studies that have examined pre-OA synovium and normal controls, it is difficult to know whether any cellular differences would be significant. Another potential confounding variable is patient heterogeneity and our relatively low sample size (*n* = 8 per group). Although our sample size is on par with a number of the studies cited within this manuscript, OA is known to be a heterogeneous disease potentially encompassing many sub-types. It is unlikely that our sample size has captured the breadth of heterogeneity observed within this disease and that must be considered as a limitation. Furthermore, since we sampled many sites within each patient (48 data points per patient) we did observe heterogeneity in marker expression (MPC and macrophage) both within and between patients. Specifically, we did observe some areas of cells positive for only one marker and others that were predominantly double positive (Figures S1 and S2); however, when this was quantified, the majority of cells (MPCs or macrophages) in all cohort were double positive for both markers (either CD90^+^ CD271^+^ or CD14^+^ CD68^+^). However, this does suggest that there are other populations in the synovium that express only one marker and it would of interest to determine if these different populations exhibit different functions.

One finding of this study that has not been discussed previously to our knowledge is the co-localization (or lack thereof) of MPCs and macrophages within the synovium. While MPCs and macrophages have both been observed throughout the synovium, in this study using CD90 and CD68 staining, it was observed that these two cell types are in close proximity to each other in normal and pre-OA synovium. While this does not directly indicate a relationship between the two cell types, it has been previously demonstrated the synovial macrophages can regulate the chondrogenic and inflammatory state of synovial MSCs [17,18]. However, in OA synovial specimens, it was observed that MPC and macrophage populations were no longer in close proximity to each other and each population appeared to be located within distinct clusters of similar cells types with defined boarders within the synovium. It is also important to note, that this result was consistent across all the eight OA patients examined, with no clear example of MPC-macrophage intermixing observed within this cohort. While this result in itself does not suggest that macrophages and MPCs are no longer interacting with each other, this observation does merit further examination to determine if in OA, the relationship between MPCs and macrophages has been altered, if the same pathways are active in both cell types (e.g., growth factors/cytokines), and whether the added distance between the cells affected the ability of each cell type to effectively communicate with the other.

Recently, studies have begun to dissect the relationship between MSCs and macrophages in the joint environment [17,18]. While there is still not direct evidence that endogenous MSCs can repair cartilage defects in humans, it is clear that these synovial MSCs/MPCs present with increased chondrogenic capacity compared to their bone marrow-derived relatives. Additionally, since it is known that macrophages can secrete factors that trigger chondrocytes to undergo apoptosis (such as Inducible nitric oxide synthase (iNOS)), this highlights the macrophage as a potential signaling ‘lynchpin’ in the joint environment that is not only capable for inducing degeneration (through chondrocyte cell death), but potentially regulating the repair capacity of local MSCs. While this study did not functionally test this hypothesis, to our knowledge we are the first to demonstrate that, in addition to the numbers of cells (MPCs, macrophages) present in the synovium at any given stage of the disease, the localization and interaction of the cells with each other may play a role in the loss of homeostasis in the arthritic joint as it progresses from pre- to post-radiographic OA.

## 4. Methods

### 4.1. Ethics Statement

Informed consent to participate was obtained by written agreement. The study protocol was approved by the University of Calgary Research Ethics Board (University of Calgary ethics #21987).

### 4.2. Subjects

A total of 24 human subjects were used for the current study. Synovial biopsies were harvested from normal (*n* = 8), pre-OA (*n* = 8) and osteoarthritic (*n* = 8) knees. Normal subjects ranged in age from 32 years to 80 years of age; pre-OA subjects had an age range of 37 years to 66 years, while patients OA had an age range of 51–82 (Table 1).

Normal control tissue samples were obtained from the Southern Alberta Tissue Donation Program. Criteria for control cadaveric donations were: no history of arthritis, joint injury or surgery (including visual inspection of the cartilage surfaces during recovery), no prescription anti-inflammatory medications, no co-morbidities (such as diabetes/cancer), and availability within 4 h of death. A minimum of four synovial biopsies (total) were taken by the recovery team from the medial and lateral compartments of the joint adjacent to the capsule [16], and care was taken to avoid the fat pad.

Synovial biopsies from pre-OA subjects were taken during scheduled diagnostic arthroscopic surgery. Surgery was performed for numerous reasons, including low grade pain, clicking or crepitus. The inclusion criterion was the absence of radiographic OA defined as a Kellgren Lawrence (K/L) grade of 0 or 1, but with an Outerbridge score of 1 or 2 based on arthroscopic examination performed by an orthopedic surgeon at the University of Calgary. Synovial biopsies from OA subjects were taken during arthroscopic surgery. The inclusion criteria for OA was meeting the American College of Rheumatology (ACR) criteria with a K/L grade of 3 or 4. Based on ethics approval from the University of Calgary, a maximum of four synovial biopsies were obtained from OA patients and these were collected from the medial and lateral compartments of the joint adjacent to the capsule.

### 4.3. Tissue Fixation and Processing

Synovium from each individual patient was fixed in 4% paraformaldahyde (PFA) at 4 °C overnight, rinsed in phosphate-buffered saline, infiltrated using an automatic tissue processor (Leica Biosystems, Wetzlar, Germany), and embedded in paraffin wax. Paraffin blocks were sectioned at a thickness of 7 μm sections using a rotary microtome (Leica RM2255). A minimum of 12 slides (48 serial sections) were cut from each block.

### 4.4. Staining

Slides were stained with hematoxylin and eosin (H&E) and by immunofluorescence (IF). For IF, sections (7 μm) were deparaffinized in CitriSolv (Fisher Scientific; Fairlawn, NJ, USA) and rehydrated through a series of graded ethanol to distilled water steps. Antigen retrieval (10 mM sodium citrate, pH 6.0, Sigma-Aldrich; St. Louis, Missouri, USA) and blocking (1:500 dilution; 100 μL goat serum: 50 mL TRIS-buffered saline, 0.1% Tween 20 (TBST) for 1 h), steps were performed prior to going through sequential wash (TBST) and the application of primary antibody. Primary antibodies conjugated to fluorophores included: CD14-FITC (Macrophage), CD68-PE (Macrophage), CD90-Alexa488 (MPC) or CD271-Alexa568 (MPC) (all BD Biosciences; Franklin Lakes, NJ, USA), and all slides were counterstained with the nucleic acid stain DAPI (4′,6-diamidino-2-phenylindole) (Sigma-Aldrich; St. Louis, MS, USA) and mounted using FluorSave reagent (Calbiochem; Darmstadt, Germany). Isotype controls for fluorescein isothiocyanate (FITC) and phycoerythrin (PE) demonstrated little to no reactivity (Figure S5).

Imaging: Slides were imaged using a Plan-Apochromat objective (20×/0.8 M27) on an Axio Scan.Z1 Slide Scanner microscope (Carl Zeiss; Oberkochen, Germany); DAPI (excitation 353 nm, emission 465 nm), Alexa488 (excitation 493 nm, emission 517 nm), and Alexa568 (excitation 565 nm, emission 576 nm).

### 4.5. Evaluation/Cell Counting

Counts of cell profiles staining positive for each antibody marker were made for each section. Each section was examined at low magnification (approximately 2×) for the purpose of choosing four random areas, two from the outer layer of the synovium (subintima) and two from the inner layer (adjacent to the synovial fluid) of the synovium (intima). This allowed for examination of two areas on the periphery and two in the central area of the tissue. Counting was performed at 40× magnification which resulted in a field of area of 35 mm^2^. Twelve sections per synovial biopsy were examined using this methodology with 4 fields of view examined per section (2 intima, 2 subintima), therefore, 48 data points were generated per patient. Positive cell counts are presented as the percentage of marker positive cells over the total number of cells (DAPI) in a 35 mm^2^ field of view. Subjective scoring of synovitis was performed on H&E stained synovium using the scale of Krenn et al. [31]. Briefly, three features of synovitis: enlargement of lining cell layer, cellular density of synovial stroma and leukocytic infiltrate were evaluated from 0—absent, to 3—strong and each feature was graded separately. The sum provided the synovitis score, which was interpreted as: 0–1, no synovitis; 2–4, low-grade synovitis; 5–9, high-grade synovitis. Grading was performed by three individuals in total, 2 were blinded.

### 4.6. Analysis

To determine if cells were single and double positive for a given marker, image analysis was performed in the Zen software suite (Carl Zeiss; Oberkochen, Germany). DAPI-positive cells that expressed at least one marker were identified and marked. The signal intensity of the FITC and PE channels was quantified after autofluorescence correction and if a cell demonstrated a signal intensity in FITC or PE of greater than 95% of the background level (based on an area intensity histogram) it was called positive for that marker. Clear areas of intense autofluorescence (e.g., blood cells) and/or process/staining artefacts were excluded from the analysis.

All statistics were performed in GraphPad Prism 6.05 (La Jolla, CA, USA). A two-way analysis of variance was used to determine if significant differences existed between the mean numbers of cell profiles for each cell type. Comparisons were made both within a joint, as well as between osteoarthritic and normal joints. A Bonferroni analysis was used for all post-hoc analyses. Alpha was set at 0.05.

## 5. Conclusions

In the current study, we identified that synovial macrophages are decreased in pre-OA joints vs. normal joints and that both MPCs and macrophages are increased in the OA synovium compared to normal and pre-OA. Furthermore, we have also demonstrated that while MPCs and macrophages are in close proximity to each other in normal and pre-OA synovium, this is not the case in OA, with both cell populations being clearly separated. While some of these results are incongruent with previous reports based on OA samples, this study suggests that a threshold level in the progression of OA needs to be breached before populations of MPCs and macrophages cells respond through expansion, infiltration and/or migration in the joint. Therefore, the functional relationship between synovial MSCs/MPCs and macrophages should be examined in greater detail to determine if this loss of co-localization in the OA synovium impacts the normal functioning of these cells and potentially contributes to the pathogenies of the disease.

## Figures and Tables

**Table 1:** Patient information: Disease state, age, sex and inflammation of synovium.

Disease State	Age Range	Sex	Krenn Score Range
Normal	32–80 (Mean 61.5)	6 M, 2 F	0–3 (Mean 1)
Pre-OA	37–66 (Mean 53.2)	6 M, 2 F	0–6 (Mean 3.4)
OA	51–82 (Mean 58.2)	5 M, 3 F	6–10 (Mean 7.9)

## Supplementary Materials

Supplementary materials can be found at www.mdpi.com/1422-0067/18/4/774/s1.

## Abbreviations

OA	Osteoarthritis
MSC	Mesenchymal Stem Cell
SF	Synovial Fluid
IL	Interleukin
TNF	Tumor Necrosis Factor
PFA	Paraformaldahyde
H&E	Hematoxylin and Eosin
TBST	TRIS-buffered saline, Tween 20
FITC	Fluorescein isothiocyanate
PE	Phycoerythrin
DAPI	4′,6-diamidino-2-phenylindole
RA	Rheumatoid Arthritis
