# Supplementary material for: Enumeration and Localization of Mesenchymal Progenitor Cells and Macrophages in Synovium from Normal Individuals and Patients with Pre-Osteoarthritis or Clinically Diagnosed Osteoarthritis
Source: Int J Mol Sci. 2017 Apr 5;18(4):774. doi: 10.3390/ijms18040774 (PMC5412358; doi:10.3390/ijms18040774)
Supplement: Supplementary file 1 [file ijms-18-00774-s001.pdf]

Supplementary Materials: Enumeration and Localization of Mesenchymal Progenitor Cells and Macrophages in Synovium from Normal Individuals and Patients with Pre-Osteoarthritis or Clinically Diagnosed Osteoarthritis

Kate O'Brien, Pankaj Tailor, Catherine Leonard, Lisa M DiFrancesco, David A Hart, John R Matyas, Cyril B Frank and Roman J Krawetz

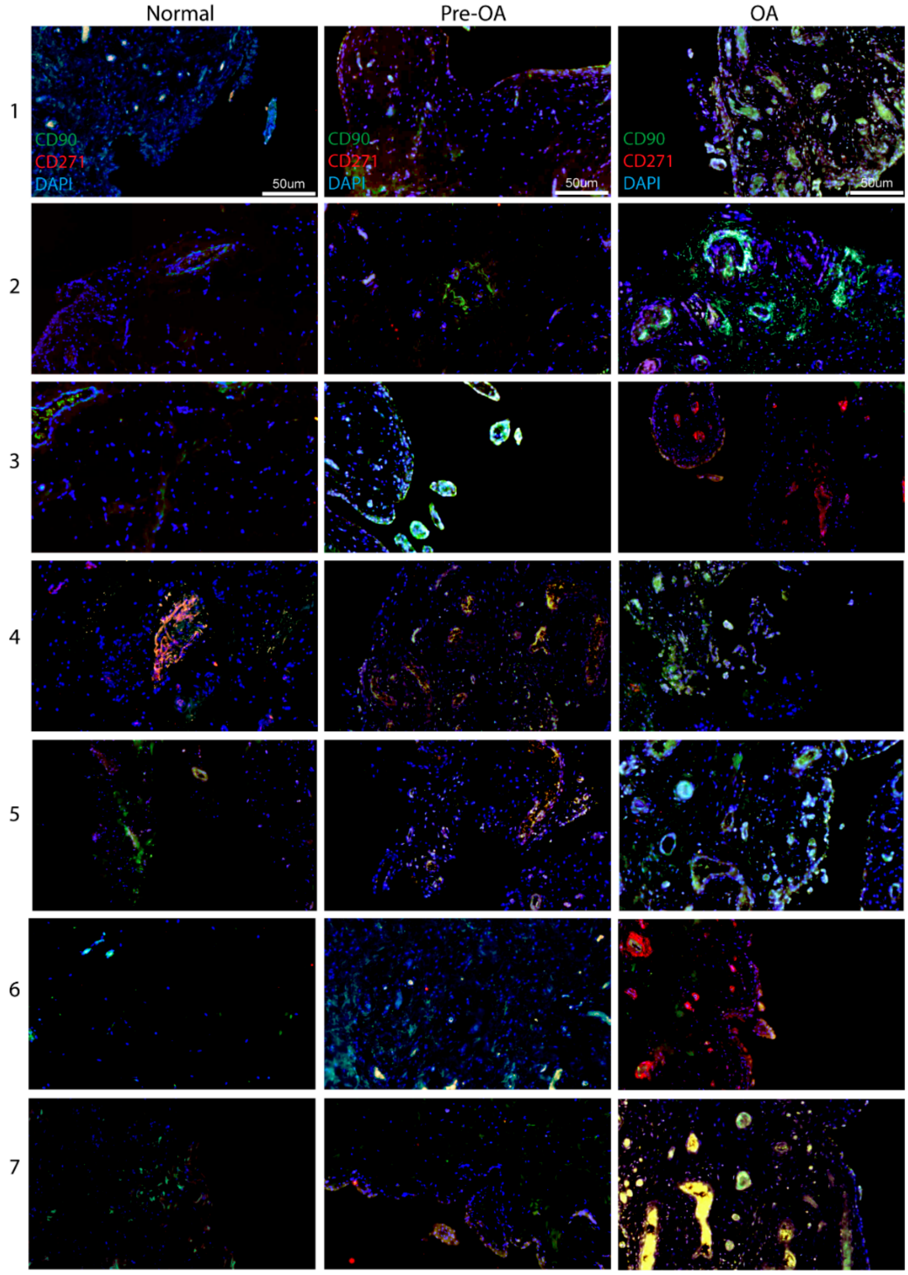

Figure S1. Markers of mesenchymal progenitor cells (MPCs) (CD90, CD271) in Normal, Pre-osteoarthritis (OA) and OA synovial tissue. Representative images from the remaining seven individuals from the normal, pre-OA and OA cohorts stained with CD90 (green), CD271 (red) and 4',6-diamidino-2-phenylindole (DAPI) (blue). Scale bar represents 50µm.

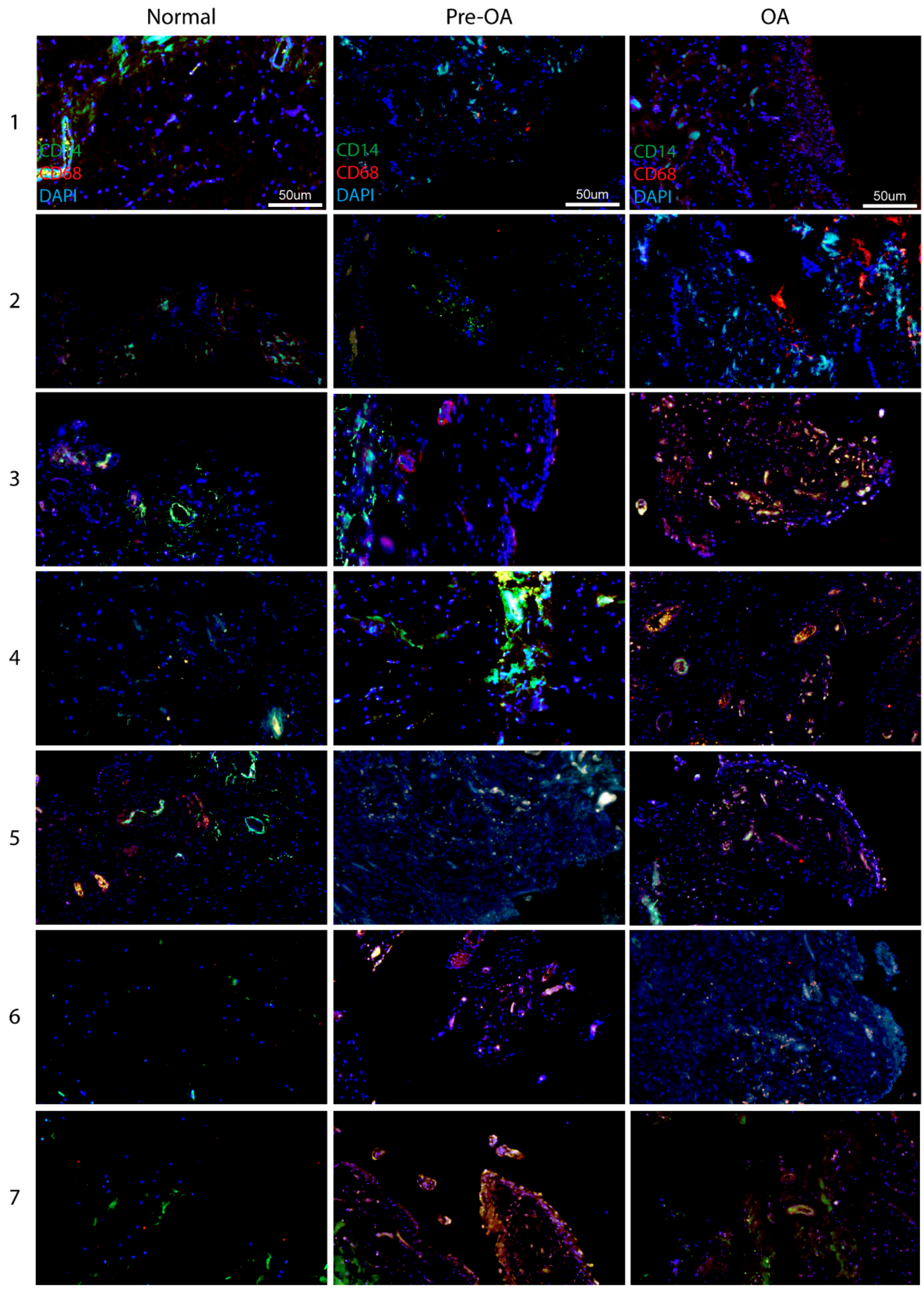

Figure S2. Markers of macrophages (CD14, CD68) in Normal, Pre-OA and OA synovial tissue. Representative images from the remaining seven individuals from the normal, pre-OA and OA cohorts stained with CD14 (green), CD68 (red) and DAPI (blue). Scale bar represents 50µm.

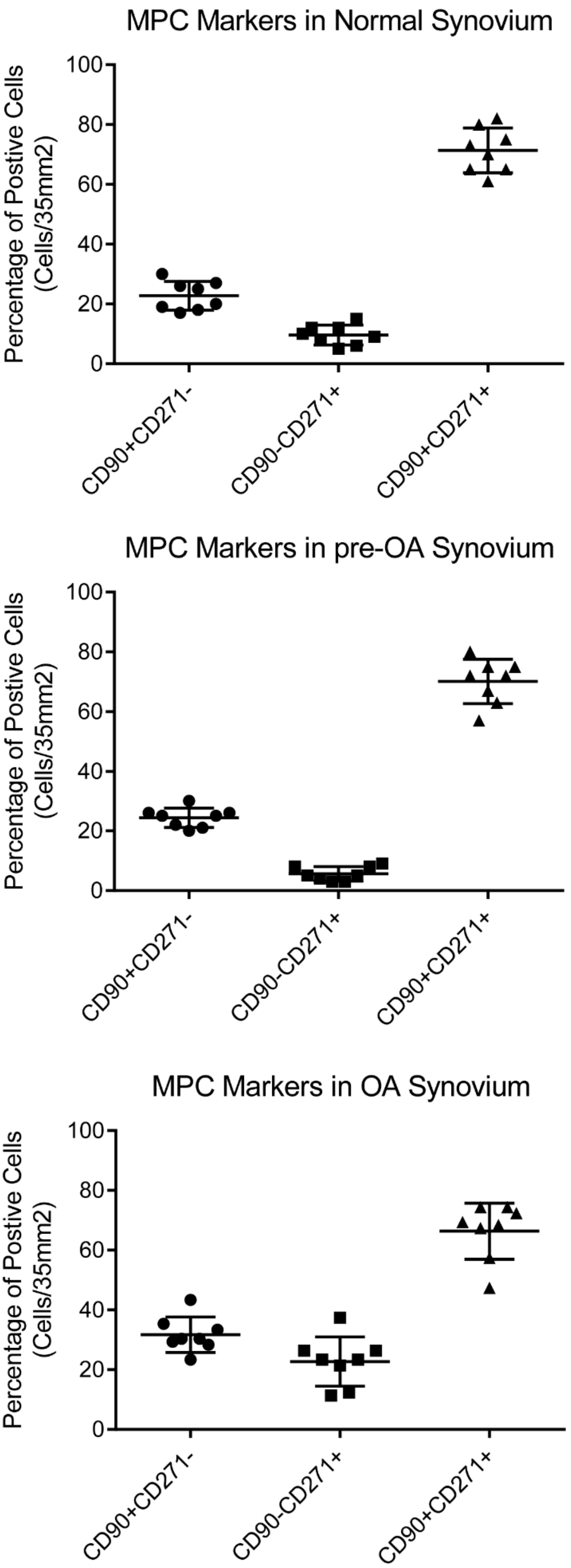

Figure S3. Enumeration of single and double positive cells expressing MPC markers. CD90+, CD271+ and CD90+ CD271+ cells were quantified in normal, pre-OA and OA synovium. In all cohorts examined, the majority of cells expressing both markers (CD90 and CD271).

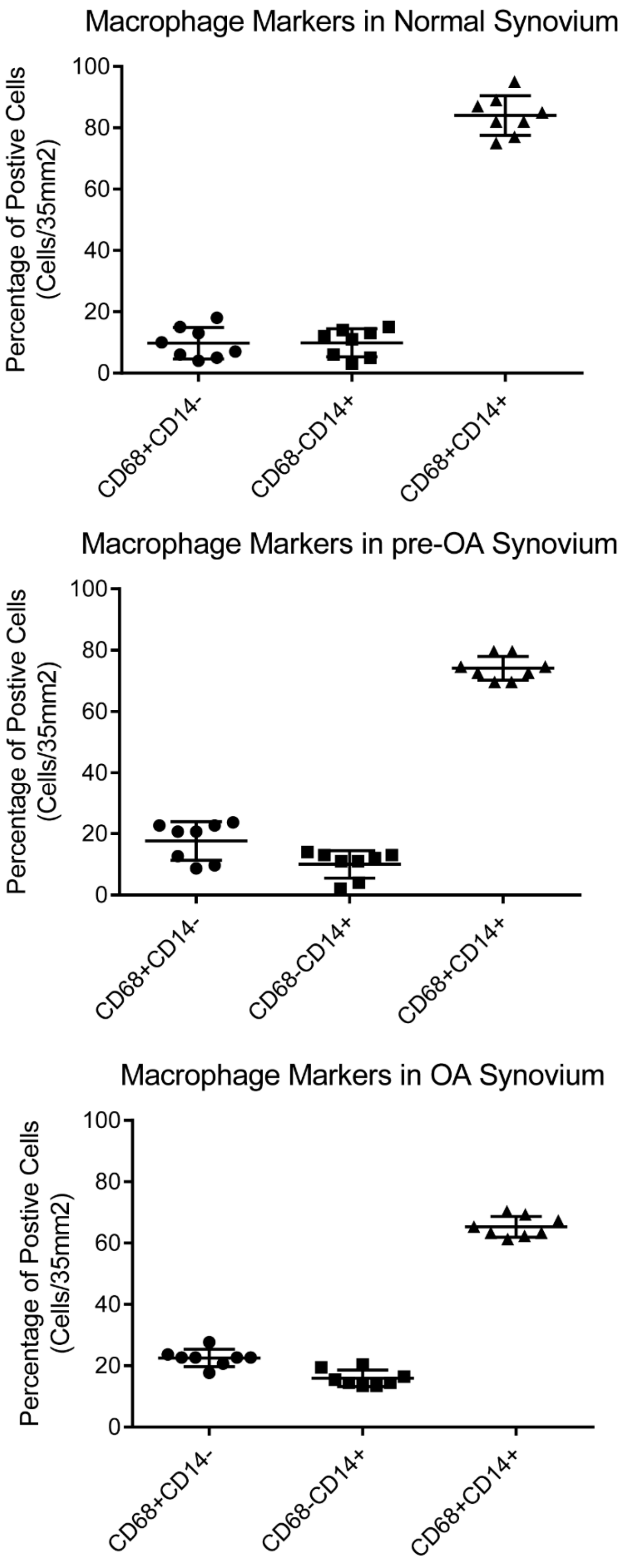

Figure S4. Enumeration of single and double positive cells expressing macrophage markers. $CD14^+$, $CD68^+$ and $CD14^+$ $CD68^+$ cells were quantified in normal, pre-OA and OA synovium. In all cohorts examined the majority of cells expressed both markers (CD14 and CD68).

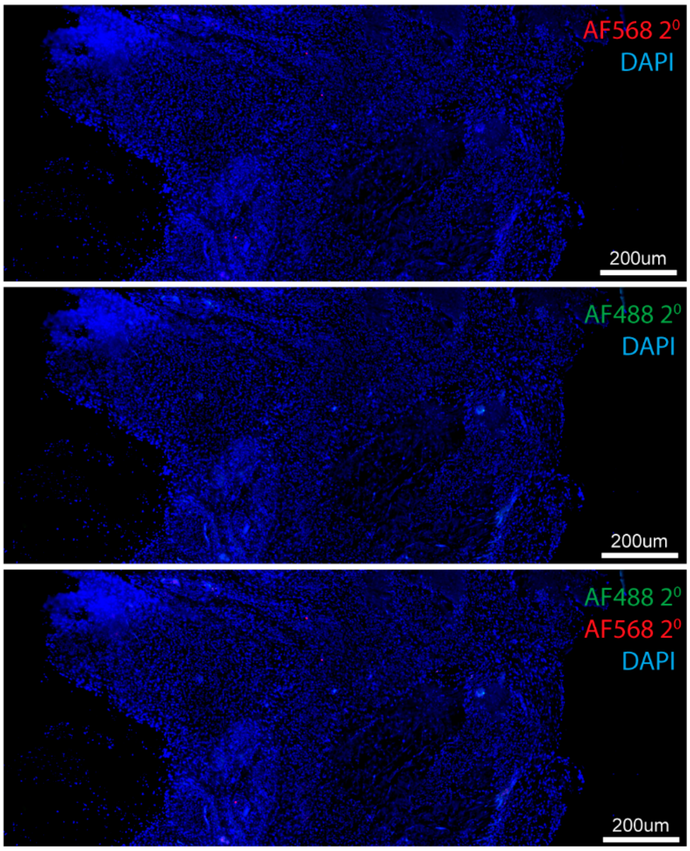

Figure S5. Isotype Controls. To control for non-specific binding of the primary antibody, isotype specific controls conjugated to each fluorophore were examined on synovial tissue sections. Limited/no reactivity was observed.
